# Diagnostic Procedures, Diagnoses, and Treatment Outcomes of Patients with Presumptive Tuberculosis Pleural Effusion in Uzbekistan

**DOI:** 10.3390/ijerph18115769

**Published:** 2021-05-27

**Authors:** Fazlkhan Abdugapparov, Ruzanna Grigoryan, Nargiza Parpieva, Sherali Massavirov, Anvar Riskiyev, Jamshid Gadoev, Mariana Buziashvili, Nestani Tukvadze, Arax Hovhannesyan, Andrei Dadu

**Affiliations:** 1 Department of Phthisiology and Pulmonology, Tashkent Medical Academy, Farabi 2, Tashkent 100109, Uzbekistan; tbcenter.uz@mail.ru; 2 Tuberculosis Research and Prevention Center, NGO, Yerevan 0023, Armenia; ruzanna.grigory@gmail.com; 3 Republican Specialized Scientific Practical Medical Center of Phthisiology and Pulmonology under Ministry of Health of the Republic of Uzbekistan, Sh. Alimov 1, Little Ring Road, Tashkent 100086, Uzbekistan; nargizaparpieva@gmail.com (N.P.); anvar_6070@list.ru (A.R.); 4 World Health Organization Country Office in Uzbekistan, 16 Tarobiy Str., Tashkent 100100, Uzbekistan; gadoevj@who.int; 5 Department of Scientific Research, National Center for Tuberculosis and Lung Diseases, 8 Adjara Str., 0101 Tbilisi, Georgia; buziashvili.mari@gmail.com (M.B.); marikushane@yahoo.com (N.T.); 6 World Health Organization Regional Office for Europe, UN City, Marmorvej 51, DK-2100 Copenhagen, Denmark; hovhannesyana@who.int (A.H.); dadua@who.int (A.D.)

**Keywords:** tuberculosis, pleural effusion, diagnosis, pleural fluid aspiration, pleural biopsy, treatment outcome, SORT-IT, operational research, Uzbekistan

## Abstract

Tuberculosis (TB) pleural effusion (TPE) is the second most common manifestation of extrapulmonary TB (EPTB), which remains a great diagnostic challenge worldwide. In Uzbekistan, there has been no formal evaluation of the actual practices of diagnosing and treating TPE. Our cohort study therefore aimed to describe the frequency and types of different diagnostic procedures of TPE during 2017–2018 and assess the association of baseline characteristics and establish diagnostic methods with TB treatment outcomes. In total, 187 patients with presumptive TPE were assessed, and 149 had a confirmed diagnosis of TPE (other diagnoses included cancer *n* = 8, pneumonia *n* = 17, and 13 cases were unspecified). TB was bacteriologically confirmed in 22 (14.8%), cytologically confirmed in 64 (43.0%), and histologically confirmed in 16 (10.7%) patients. Hepatitis was the only co-morbidity significantly associated with unsuccessful treatment outcomes (RR 4.8; 95%CI: 1.44–15.98, *p* value 0.011). Multivariable regression analysis showed that drug-resistant TB was independently associated with unsuccessful TB treatment outcome. (RR 3.83; 95%CI: 1.05–14.02, *p* value 0.04). Multidisciplinary approaches are required to maximize the diagnostic accuracy of TPE and minimize the chances of misdiagnosis. TPE patients with co-infections and those with drug resistance should be more closely monitored to try and ensure successful TB treatment outcomes.

## 1. Introduction

Tuberculosis (TB), caused by *Mycobacterium tuberculosis* (MTB), remains one of the top ten causes of death around the world and a leading cause of death from infectious disease. According to World Health Organization (WHO) estimates, 10 million people developed TB and 1.4 million died from the disease in 2019 [1]. While MTB can affect almost all organs and tissues, pulmonary disease accounts for the majority of TB cases. Other forms of TB are categorized as extrapulmonary TB (EPTB) and, while less common than pulmonary TB, these types also significantly contribute to the burden of disease, ranging from 15% to 25% of all TB cases across countries [2,3,4]. About 16% of the 7.1 million incident TB cases in 2019 were EPTB [1].

TB is one of the known and important causes of pleural effusion, especially in countries with a high prevalence of TB [3]. TB pleural effusion (TPE) is the second most common manifestation of EPTB [5,6]. Pleural TB remains a great diagnostic challenge since conventional smear examination for acid-fast bacilli (AFB) and mycobacterial culture have poor sensitivity in pleural fluid [7].

The TB incidence remains high in the countries of Central Asia. In 2019, the TB case notification rate in Uzbekistan (one of the countries of Central Asia) was 49.3 per 100,000 population, while the EPTB case notification rate was 17.9 per 100,000 population. The treatment success rate for patients with new and relapsed TB is reported at 92% by the National TB Program (NTP) [1], but there are no official statistics or routine reports on the treatment outcomes of patients with pleural effusion (including TPE) in the country.

The WHO has developed special programs with detailed recommendations and guidelines for diagnosing and treating pulmonary and extrapulmonary TB, including TPE [8,9]. Despite the increasing case notification rates of EPTB [4,10], there has been a limited focus on this condition, and targeted interventions to respond to the needs of patients with different types of EPTB remain inadequate. The WHO currently recommends that TPE is managed according to the general diagnostic and treatment guidelines that are available for the category of EPTB as a whole. Despite smear microscopy for AFB, mycobacterial culture, and Xpert MTB/RIF Ultra showing low sensitivity in pleural aspirates, these remain the main tools for bacteriological confirmation of TPE [8,9]. Where possible, these tests are complemented by pleural biopsy with the aim of looking for histological changes that indicate infection with MTB [8,11]. In many cases, the diagnosis of TPE is based on clinical judgment that is supported by conditional evidence-based laboratory measurements that include white blood cells, protein content, and adenosine deaminase (ADA) in the pleural fluid [3,5].

Although the Ministry of Health of Uzbekistan has established an algorithm for the diagnosis and treatment of presumptive TPE which is in line with the WHO recommendations [8], there has been no formal evaluation of actual practices of diagnosing and treating TPE in Uzbekistan. To address this gap, our study aimed to describe the frequency and types of different diagnostic procedures performed in patients with presumptive TPE registered at the Republican Specialized Scientific Practical Medical Center of Phthisiology and Pulmonology (RSSPMCPP) in Tashkent, Uzbekistan, between 2017 and 2018 and to assess predictors associated with TB treatment outcome in those with diagnosed TPE.

## 2. Materials and Methods

### 2.1. Study Design

This was a cohort study using secondary data from medical charts and TB forms of patients with presumptive TB pleural effusion referred to the inpatient department of RSSPMCPP in Tashkent, Uzbekistan, from 2017 to 2018.

### 2.2. Study Setting—General, Study Site, and Study Period

Uzbekistan is a landlocked country located in Central Asia surrounded by Afghanistan, Kazakhstan, Kyrgyzstan, Tajikistan, and Turkmenistan. It has a population of about 32 million—about 50% of the whole of Central Asia’s population. Uzbekistan’s income level was upgraded from a low- to a lower middle-income country in 2011. However, according to 2015 data, about 13% of the population still live below the national poverty line [12].

The TB care in Uzbekistan is implemented and coordinated by the National TB Program through the efforts of the RSSPMCPP. All activities related to TB diagnosis and treatment are provided free of charge. TB care is provided throughout all levels of the health care system. The first level represents primary health care facilities (polyclinics), and this is the usual entry point for patients with presumptive TB. The second level of TB care is provided at the district level in TB clinics where patients undergo initial examinations including laboratory tests, microscopy, XpertMTB/RIF assays, and X-ray. Patients may be further referred to the third level of TB care for additional laboratory examinations including mycobacterial culture and Mycobacteria Growth Indicator Tube (MGIT) and inpatient care. The fourth level of TB care is provided in the RSSPMCPP, where patients with complicated diagnoses and advanced co-morbidities are seen and managed. Patients with presumptive TPE usually enter the first and second levels of TB care and are further referred to the third and the fourth level of TB care for more precise diagnosis and treatment. TPE patients who are found to be sputum smear-positive are classified as pulmonary TB cases and receive TB care for pulmonary TB on an inpatient level. The national TB treatment protocols are administered in accordance with the WHO guidelines [8]. The same standard treatment regimens are used for patients with both pulmonary TB and EPTB.

### 2.3. Study Population

All patients with presumptive TPE admitted to the inpatient department of RSSPMCPP from 2017 to 2018 were included in the study. The final diagnosis was made based on clinical manifestations, radiological evaluation (chest X-ray/ultrasound/chest CT scan), and examination of the extracted pleural fluid: bacteriological (smear microscopy for AFB, Xpert MTB/RIF, and mycobacterial culture) and/or cytological and biochemical examinations as well as histological evaluation of the pleural biopsy.

### 2.4. Sources of Data

Data were extracted from the patient’s TB forms and inpatient medical charts available at the RSSPMCPP.

### 2.5. Data Collection and Validation

Data extracted from the patients’ medical charts and patient TB forms were entered into standard electronic records which were developed using the EpiData application (version 3.1 EpiData Association, Odense, Denmark). Data were checked for errors and discrepancies using cross-tabulation and analysis of extreme values. Inconsistencies were resolved by retrieving the source documents.

### 2.6. Data Variables

Demographic, socio-economic, and medical history-related variables that were considered appropriate for achieving the study objectives were as follows: age, sex, place of residence, tobacco use, alcohol use, co-morbidities (diabetes mellitus, human immunodeficiency virus (HIV), hepatitis C virus (HCV)), known diagnosis of TPE, drug-resistance profile, treatment information and outcome, details on sputum and pleural fluid examination (microscopy, Xpert MTB/RIF, mycobacterial culture, cytology), and histology.

#### Definitions

Diabetes status was identified based on clinical history or blood glucose measurement followed by an endocrinologist’s evaluation. All patients were tested for HIV and HCV antibodies at baseline and positive rapid test results were further confirmed. Data on smoking and alcohol use were obtained based on patient self-reports. Tuberculosis treatment outcomes were classified according to the WHO recommended definitions: successful outcome (including cure and treatment completion) and unsuccessful treatment outcome comprising failure, loss to follow-up, and death [13].

### 2.7. Analysis and Statistics

Descriptive statistics were used to describe patient demographic and clinical characteristics. These included: frequencies, proportions, measures of central tendency (mean), and variation (standard deviation). Differences between the groups were assessed with the use of Pearson’s χ^2^ test (Chi square) for categorical variables. The level of significance was set at *p* < 0.05. Risk ratios (RR) as a measure of association between predictors and treatment outcome (unfavorable vs. favorable) were calculated using binomial log-linear regression and presented in respective tables. Along with the parameter estimates, 95% confidence intervals (CI) and *p* values were calculated as well. Analysis was performed using Intercooled Stata software version 15 (Stata Corp., College Station, TX, USA).

## 3. Results

**Figure 1:**
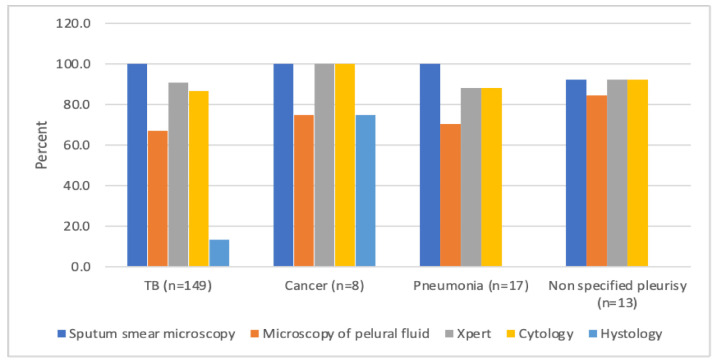
The proportions of different diagnostic procedures undertaken in patients with pleural effusion in relation to the final diagnosis in the Republican Specialized Scientific Practical Medical Center of Phthisiology and Pulmonology (RSSPMCPP), Tashkent, Uzbekistan, 2017–2018.

In total, 187 patients with presumptive TPE were examined with different diagnostic procedures. Figure 1 shows the proportions of diagnostic procedures performed in relation to the final diagnosis of the study population.

Among 187 patients with bacteriological examinations, cytology, and histology tests, 149 (79.7%) were diagnosed with tuberculosis pleural effusion. Eight (4.3%) patients had cancer, 17 (9.1%) patients had pneumonia, and 13 (6.9%) cases remained unspecified. Thus, 38 patients were excluded from the final analysis which focused on those with diagnosed TPE.

Overall, sputum smear microscopy was performed in all 149 (100%) patients and Xpert MTB/RIF was performed in 135 (90.6%) patients. Smear microscopy of pleural fluid was performed in 100 (67%) patients, cytological tests in 129 (86.6%), and histological tests in 20 (13.4%).

Among 149 patients with confirmed TPE, 89 (59.7%) were male; mean age was 37.9 (SD 11.1), and the majority of the patients resided in rural areas of the country (*n* = 108, 72.5%). The main reported comorbidities were arterial hypertension (*n* = 9, 47.4%), coronary heart disease (*n* = 5, 26.3%), hepatitis (3.4%), and HIV (2.0%). There were a few other rare reported co-morbidities that included lymphoma (*n* = 1, 5.3%), epilepsy (*n* = 1, 5.3%), chronic cholecystitis (*n* = 1, 5.3%), and pyelonephritis (*n* = 1, 5.3%). Thirty-seven (24.8%) patients were reported to be tobacco users and 9 (6.0%) were alcohol users.

TB was bacteriologically confirmed in 22 (14.8%) patients: 20 (13.4%) patients had positive MTB result with Xpert MTB/RIF test performed from sputum and pleural fluid; 6 patients (4%) had a positive acid-fast bacilli (AFB) result with microscopy (5 (3.4%) from pleural fluid and one patient (0.7%) from sputum). The yield of pleural TB was highest for histological examination (61.5%, *n* = 16), followed by cytological examination (39.0%, *n* = 64) and Xpert MTB/RIF (11.8%, *n* = 20). In 72 (48.3%) patients diagnosed with TPE, bacteriological, histological, and cytological tests were either negative or not done. Table 1 shows details of the yield of the performed tests.

The majority of patients with diagnosed TPE had a successful treatment outcome (*n* = 135, 91%). There was no association between age or gender and risk of unfavorable treatment outcome.

Six (8.7%) patients were confirmed to have resistance to at least rifampicin, and having drug-resistant TB significantly increased the risk of unsuccessful treatment outcomes (RR 3.97; 95% CI: 1.13–13.93, *p* value 0.031). Hepatitis was the only co-morbidity to be significantly associated with unsuccessful treatment outcomes (RR 4.8; 95% CI: 1.44–15.98, *p* value 0.011). There were no significant associations between TB treatment outcome and (i) the different diagnostic procedures performed or (ii) whether the TB diagnosis was confirmed by bacteriological/histological methods (Table 2).

In the final multivariable regression analysis, only drug resistance remained a significant risk factor for completing TB treatment unsuccessfully (RR 3.83; 95% CI: 1.05–14.02, *p* value 0.04).

## 4. Discussion

The diagnosis of pleural TB is still a challenge worldwide due to insensitive laboratory detection test results [14,15]. However, an accurate and timely diagnosis of TPE remains essential for achieving a successful treatment outcome among these patients.

Our findings demonstrate that overall, all the diagnostic procedures including bacteriological, cytological, and histological examination of pleural fluid for confirmation of TPE are commonly used in Uzbekistan and there is no one, standardized approach to diagnosing the TPE. In our study, pleural fluid and sputum smear microscopy were positive for AFB only in 3.4% and 0.7% of cases, respectively. This is relatively low compared to earlier studies from Spain showing 6% and 8% of cases having positive AFB in sputum and pleural fluid smears, respectively [16,17,18]. Among analyzed TPE patients, 13.4% were MTB positive on Xpert MTB/RIF. Although the Xpert MTB/RIF assay has long been known to be a reliable diagnostic test for EPTB, it was not actually endorsed as an initial diagnostic test for TPE until updated guidelines in 2020; this could probably be attributed to the presence of polymerase chain reaction inhibitors in pleural fluid and a low bacillary load [9,14,19,20].

In our study, 10.7% of TPE cases had a histological confirmation of TB, which was similar to a South Korean study showing that 13.9% of patients had a positive histological diagnosis of TPE [21]. Of interest, while there is little evidence in the literature about using cytological examination for diagnosing TPE, a high proportion of our study participants (43.0%) were confirmed to have TPE cytologically.

Among our study participants, eight (4.3%) were confirmed to have a malignant disease. The proportion of presumptive TPE with cancer varies from 7.3% to 55.4% across the published literature [21,22,23]. The differences between our study and the other studies are explained by the specific study populations. Our study participants were selected at specialized tuberculosis clinics and had already undergone various diagnostic procedures at the first and second levels of the health system to rule out diseases other than TB. Given the clinical and laboratory similarities (pleural fluid white cell count and protein content) between TB and malignant pleural effusions, it is essential to try and reach a timely diagnosis to avoid inappropriate treatment, especially in high TB-prevalent settings.

The majority of the patients included in our study had a successful treatment outcome (90.6%). This proportion is similar to that found in a study conducted in Barcelona, Spain (91.3%), but is better than that shown in similar cohorts from India (78.1%) and South Korea (88%) [22,24,25]. A further detailed analysis could possibly help explain a high treatment success rate, which, unfortunately, was not a part of our retrospective study. One possible explanation that we observed could be a small proportion of severe co-morbidities as well as HIV co-infection, which could be significantly decreasing the risk of unsuccessful treatment outcomes.

The risk of unsuccessful treatment outcome was significantly increased for those with hepatitis co-infection and those with drug-resistant TB (RR 4.80; 95% CI: 1.44–15.98, *p* value 0.011 and RR 3.97; 95% CI: 1.13–13.93, *p* value 0.031, respectively). These findings are consistent with studies from China and South Africa, looking at different risk factors associated with unsuccessful anti-TB treatment outcomes (*p* values 0.010 and 0.023, respectively) [26,27]. The results are not surprising, considering the hepatic disease-related impairment and possible impact on the effect of anti-TB medications.

The study has several limitations. First, this was a retrospective study with inevitable information bias. We analyzed patient charts which were inconsistently completed and/or might have had missing information, as there are no standardized data recording practices across Uzbekistan clinics. Second, the uncommon occurrence of TPE among the general TB population led to a small sample size, which could have interfered with a robust analysis of factors associated with unfavorable treatment outcomes. Third, the study did not assess measurements of pleural fluid adenosine deaminase (ADA), a well-known biomarker for TPE, as the test is not included in the routine practice in Uzbekistan. The ADA has been long considered a highly accurate marker for diagnosing TPE and, especially, in moderate to high TB burden countries like Uzbekistan, the detection of high ADA levels can help confirm TB diagnosis and justify the timely initiation of relevant treatment. [16,21,22,28].

## 5. Conclusions

Due to the lack of highly sensitive methods, the diagnosis of pleural effusion remains a challenge. Multidisciplinary approaches are required to maximize diagnostic accuracy and minimize the chances of misdiagnosis, which in turn will lead to better TB-specific treatment outcomes. An incorporation of the ADA test might improve the diagnostic approach to identifying pleural TB among Uzbekistan patients. TPE patients with concomitant infections/conditions and those with drug-resistant organisms should be more closely monitored to ensure that they successfully complete TB treatment. A detailed cost analysis of the diagnostic tests would greatly benefit the country program to assess and establish the best TPE diagnostic approach based on cost and test performance.

## Figures and Tables

**Table 1:** Positivity of the tests performed and their diagnostic value among patients with presumptive TB Pleural Effusion (*n* = 187).

Diagnostic Procedure	Total Tests	Confirmed Pleural TB Diagnosis		Confirmed Cancer Diagnosis
	*n*	*n*	(%)	
Bacteriological analysis				-
Sputum smear microscopy for AFB	186	1	(0.5)	-
Pleural fluid microscopy for AFB	129	5	(3.9)	-
Mycobacterial culture of pleural fluid	3	0	(0.0)	-
Xpert MTB/RIF (pleural fluid and/or sputum)	170	20	(11.8)	-
Cytology of pleural fluid	164	64	(39.0)	8
Histology of pleural biopsy	26	16	(61.5)	8

AFB = acid-fast bacilli.

**Table 2:** Predictors of the treatment outcome in patients with diagnosed tuberculosis pleural effusion enrolled into treatment in RSSPMCPP, Tashkent, Uzbekistan, 2017–2018.

Characteristics	Total		Successful Outcome		Unsuccessful Outcome		RR	95% CI	*p* Value
	*n*	%	*n*	(%)	*n*	(%)			
Age group									
<40 years	86	(57.7)	79	(91.9)	7	(8.1)	1		
40 years and over	63	(42.3)	56	(88.9)	7	(11.1)	1.37	(0.50–3.70)	0.540
Gender									
Male	89	(59.7)	81	(91.0)	8	(9.0)	1		
Female	60	(40.3)	54	(90.0)	6	(10.0)	1.11	(0.41–3.04)	0.836
Region of referral									
Tashkent city	30	(20.1)	29	(96.7)	1	(3.3)			
Tashkent region	36	(24.2)	31	(86.1)	5	(13.9)			
Other cities ^	83	(55.7)	75	(90.4)	8	(9.6)			
Place of residence									
Urban	41	(27.5)	37	(90.2)	4	(9.8)	1		
Rural	108	(72.5)	98	(90.7)	10	(9.3)	0.95	(0.32–2.86)	0.926
Drug-resistance									
Sensitive/not bac.confirmed	143	(96.0)	131	(91.6)	12	(8.4)	1		
Confirmed RR/MDR	6	(4.0)	4	(66.7)	2	(33.3)	3.97	(1.13–13.93)	0.031
Smoking									
Yes	37	(24.8)	35	(94.6)	2	(5.4)	1.98	(0.46–8.45)	0.355
No	112	(75.2)	100	(89.2)	12	(10.7)	1		
Alcohol use									
Yes	9	(6.0)	9	(100.0)	0	(0.0)			
No	140	(94.0)	126	(90.0)	14	(10.0)			
HIV									
Yes	3	(2.0)	2	(66.7)	1	(33.3)	0.27	(0.05–1.44)	0.124
No	146	(98.0)	133	(91.1)	13	(8.9)			
Diabetes									
Yes	3	(2.0)	2	(66.7)	1	(33.3)	3.74	(0.70–20.13)	0.124
No	146	(98.0)	133	(91.1)	13	(8.9)			
Hepatitis									
Yes	5	(3.4)	3	(60.0)	2	(40.0)	4.80	(1.44–15.98)	0.011
No	144	(96.6)	132	(91.7)	12	(8.3)	1		
Other comorbidities *									
Yes	19	(12.8)	16	(84.2)	3	(15.8)	1.87	(0.57–6.09)	0.301
No	130	(87.2)	119	(91.5)	11	(8.5)	1		

RR: Risk ratio, CI: Confidence Interval, TPE: Tuberculosis pleurisy. * Other co-morbidities included arterial hypertension (9), coronary heart disease (5), lymphoma (1), pregnancy (1), epilepsy (1), chronic cholecystitis (1), chronic pyelonephritis (1). ^ Other cities of referral included: Andijon, Bukhara, Farghona, Jizzakh, Khorazm, Namangan, Nawoiy, Qashqadaryo, Samarqand, Sirdaryo, Surkhondaryo, and Karakalpakstan Republic.

## Data Availability

The data that support the findings of this study are available from the corresponding author, [F.A.], upon reasonable request.

## Open Access Statement

In accordance with WHO’s open-access publication policy for all work funded by WHO or authored/co-authored by WHO staff members, the WHO retains the copyright of this publication through a Creative Commons Attribution IGO license (http://creativecommons.org/licenses/by/3.0/igo/legalcode accessed on 12 January 2021) which permits unrestricted use, distribution, and reproduction in any medium provided the original work is properly cited.
